# The impact of the COVID-19 pandemic on measles surveillance in the World Health Organisation African Region, 2020

**DOI:** 10.11604/pamj.2021.39.192.29491

**Published:** 2021-07-08

**Authors:** Balcha Masresha, Richard Luce, Reggis Katsande, Annick Dosseh, Patricia Tanifum, Emmaculate Lebo, Charles Byabamazima, Anfumbom Kfutwah

**Affiliations:** 1 World Health Organisation, Regional Office for Africa, Brazzaville, Congo,; 2 World Health Organisation, Inter-Country Support Team for Western Africa, Ouagadougou, Burkina Faso,; 3 World Health Organisation, Inter-Country Support Team for Central Africa, Libreville, Gabon,; 4 World Health Organisation, Inter-Country Support Team for East and Southern Africa, Harare, Zimbabwe

**Keywords:** Vaccine preventable diseases, measles surveillance, rubella, measles laboratory, COVID-19, Africa

## Abstract

**Introduction:**

following the declaration of the COVID-19 pandemic, many countries imposed restrictions on public gatherings, health workers were repurposed for COVID-19 response, and public demand for preventive health services declined due to fear of getting COVID-19 in health care settings. These factors led to the disruption in health service delivery, including childhood immunization, in the first months of the pandemic. Measles surveillance supported with laboratory confirmation, is implemented in the African Region as part of the strategies towards attaining measles elimination. World Health Organisation developed guidelines to assist countries to continue to safely provide essential health services including immunization and the surveillance of vaccine preventable diseases during the pandemic.

**Methods:**

we analysed the measles case-based surveillance and laboratory databases for the years 2014 to 2020, to determine the impact of the COVID-19 pandemic on measles surveillance, comparing the performance in 2020 against the preceding years.

**Results:**

the weekly reporting of suspected measles cases declined starting in April 2020. Twelve countries had more than 50% decline in both the number of reported cases as well as in the number of specimens collected in 2020, as compared to the mean for the years 2014-2018. In 2020, only 30% of the specimens from suspected measles cases arrived at the national laboratory within 3 days of collection. At Regional level, 86% of the districts reported suspected measles cases in 2020, while the non-measles febrile rash illness rate was 2.1 per 100,000 population, which was the lowest rate documented since 2014. Only 11 countries met the targets for the two principal surveillance performance indicators in 2020 as compared to an average of 21 countries in the years 2014-2019.

**Conclusion:**

the overall quality of measles surveillance has declined during the COVID pandemic in many countries. Countries should implement immediate and proactive measures to revitalise active surveillance for measles and monitor the quality of surveillance. We recommend that countries consider implementing specimen collection and testing methods that can facilitate timely confirmation of suspected measles cases in remote communities and areas with transportation challenges.

## Introduction

In 2011, the World Health Organization (WHO) African Regional Committee endorsed a Regional goal for measles elimination by 2020. Measles elimination is defined as the absence of endemic measles cases for a period of twelve months or more, in the presence of adequate surveillance, and when the following criteria are met: achieving and maintaining at least 95% coverage with both first dose measles vaccination and the second opportunity of measles vaccination in all districts and at the national level; and achieving a measles incidence of less than 1 confirmed measles case per million inhabitants per year [1]. The elimination goal includes, among others, the objective to improve the quality of measles surveillance, as well as the epidemiological and virological investigation of measles outbreaks in all countries [1]. This goal was adopted following successful attainment of measles control in the Region since 2001, attaining 92% reduction in estimated measles mortality by 2008 compared to 2000 [2]. The Regional Strategic plan for Immunization 2014-2020 further elaborated the strategies and set programmatic targets towards measles elimination, including the attainment of surveillance performance targets in all countries [3].

Following the rapid global spread of the SARS-COV-2 outbreak, WHO declared a pandemic on 11^th^ March 2020 [4]. The number of COVID-19 reported cases in the countries of the WHO African Region reached 303,986 cases by 30^th^ June 2020 [5]. By the end of 2020, the number had increased to a total of 1,831,227 COVID-19 cases and 40,299 deaths [6]. During the year, many countries imposed restrictions on public gatherings and travel, health workers and health facilities were repurposed for COVID-19 response, and public fear of getting COVID-19 in health care settings all contributed to the disruption in health service delivery, especially in the first 3 to 6 months of the pandemic. Modelling showed the possible effects of such disruptions on maternal and child health, unless addressed timely [7]. In the African Region, the actual decline in routine immunization service delivery as well as the postponement of scheduled preventive mass vaccination campaigns using measles, polio, yellow fever and other antigens have been documented [8,9]. Data from the WHO European region indicates a decline in measles surveillance performance in 2020, with a sharp decrease in the number of reported cases between the months of January and May 2020 [10]. World Health Organisation developed guidelines to assist countries to continue to safely plan and implement the provision of essential health services including immunization and the surveillance of vaccine preventable diseases during the pandemic [11,12].

Case-based measles surveillance, supported with laboratory confirmation, has been implemented in the African region since 2002. In this surveillance system, unless in the context of a confirmed outbreak, each suspected measles case is investigated including the collection of a blood specimen for Immunoglobulin M (IgM) serological confirmation testing. If the laboratory result is negative for measles IgM testing, then the specimen is systematically tested for rubella IgM. In addition to laboratory confirmation, measles cases are also confirmed by epidemiological linkage to laboratory confirmed cases in an outbreak setting, or confirmed by clinical compatibility if specimen was not collected when it should have been and the case has not been linked to an outbreak [13]. Each country in the surveillance network is supported by at least one officially designated measles serology laboratory, which utilizes standard test protocols and tools. Three Regional Reference Laboratories serve as external quality control testing laboratories. Both national and regional reference laboratories belong to the WHO Global Measles Rubella Laboratory Network that monitors laboratory performance as well as puts in place quality assurance measures to maintain high standards across all the laboratories. This manuscript attempts to look at the quality of measles surveillance in 2020 in comparison with performance over the preceding years, in order understand the impact of the COVID-19 pandemic on the surveillance performance.

## Methods

**The WHO measles cased-based surveillance and laboratory databases:** countries in the WHO Africa region are required, as part of their measles surveillance activities, to collect data for every single measles suspected case. The case-based dataset includes as key variables, the demographic and epidemiological information, laboratory confirmation and vaccination status for each suspected measles case. These records are compiled, at the national level, into a computerized database that is shared with WHO on a weekly basis. In addition, the national serological laboratories across the Region update their measles-rubella laboratory database and share it weekly with WHO. Surveillance performance is monitored monthly and summarised annually using multiple standard indicators, of which two are considered the key monitoring indicators and are tracked at the WHO Regional level. The non-measles febrile rash illness rate measures the level of case finding and investigation even in the absence of measles cases and outbreaks. The proportion of districts investigating suspected measles cases with blood specimen provides a measure of the representativeness of subnational administrative units in the case investigation efforts.

**Study type and settings:** this study is a desk review of the WHO AFRO measles case-based surveillance database covering seven years (2014 to 2020).

**Data analysis:** we analysed the case-based surveillance data for the 7 years period of the regional strategic plan for immunization (2014 to 2020), to determine the likely impact of the COVID-19 pandemic on measles surveillance in 2020, comparing the performance in 2020 against the preceding years. The temporal trends (2014-2020) of key measles case-based surveillance indicators was assessed for any significant changes potentially attributable to the disruptions of health service (including measles surveillance) caused by the COVID-19 pandemic. We also reviewed the measles-rubella national laboratory database to compare the performance indicators over the same period.

## Results

**Figure 1:**
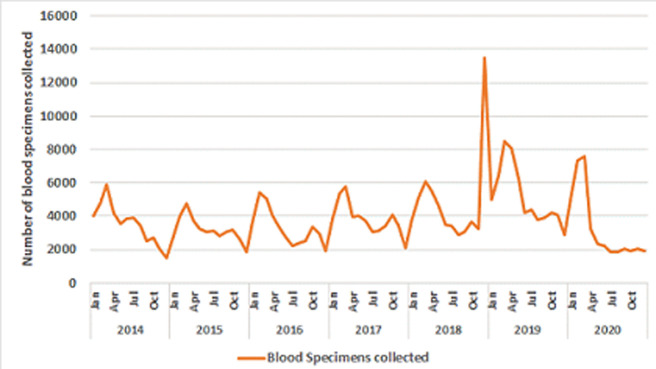
monthly number of blood specimens collected from suspected measles cases for laboratory confirmation; measles laboratory surveillance database 2014-2020

**Trends in the reporting and laboratory investigation of suspected measles cases:** the average number of suspected measles cases reported annually through the case-based surveillance system in the years 2014-2018 was 68,299 cases (ranging from 61,771 in 2016 to 82,805 in 2018). In 2020, a total of 70,242 suspected measles cases were reported throughout the Region. The largest number of suspected measles cases (323,424 cases) was reported in 2019, and it was 4 -5 times the average for preceding years. This increase was mainly due to the outbreaks in 3 countries (Madagascar, Democratic Republic of Congo and Nigeria), which accounted for 84% of the total case reports in the Region. Eighty three percent of the suspected measles cases reported in 2020 have benefited from investigation (laboratory testing or appropriate epidemiological linkage), including 39,613 blood specimens collected from suspected measles cases across the 47 countries in the Region. The average number of blood specimens collected from suspected measles cases during this 7-years period was 45,517 per year, with an average of 20% being positive for measles IgM serology testing. The highest proportion of IgM positivity for measles was documented in 2020, where 31% of all tested specimens were positive for measles (Table 1). Excluding the outbreak year of 2019, there were an average of 6710 laboratory confirmed measles cases from 2014 to 2018, while in 2020, a total of 11,781 measles cases were confirmed by laboratory. The number of laboratory confirmed rubella cases declined in 2020, with only 2848 rubella IgM positive cases, as compared to an average of 4651 for the years 2014-2018 Table 1. Comparing the number of suspected cases and the specimens collected in 2020 against the mean from each country in the years 2014-2018, we note that 12 countries (Benin, Botswana, Congo, Gabon, Malawi, Mauritius, Namibia, Sierra Leone, South Africa, Togo, Uganda and Zimbabwe) had more than 50% decline in both the number of reported cases as well as in the number of specimens collected.On the other hand, 11 countries (Algeria, Angola, Eritrea, Eswatini, Gambia, Kenya, Mauritania, Mauritius, Senegal, Seychelles, Zambia) had a decrease of between 10-50% in the number of suspected cases reported while 3 countries had less than a 5% change. Nigeria and South Sudan had an increase in the number of suspected measles cases but a decrease in the number of specimens collected, while Angola had a decrease in the number of suspected cases, but an increase in the number of samples collected Table 2. Fifteen countries (Burkina Faso, Burundi, Central African Republic, Chad, Comoros, Côte d’Ivoire, Democratic Republic of Congo, Ghana, Guinea, Liberia, Mali, Mozambique, Niger, Rwanda and Tanzania) reported more cases and also collected more specimens for serological testing in 2020 as compared to the average for 2014-2018 (Table 2). The monthly trends of reporting of suspected measles cases across the Region for 2020 indicates a marked reduction starting in April. The lowest number of reported cases were in July and in December respectively, when less than 2000 suspected cases were reported monthly from the whole Region. A similar decline was seen in the number of laboratory specimens collected starting in the month of April, with the lowest number in July 2020 (Figure 1). More than two thirds of the monthly reported cases had blood specimens collected in the second half of 2020, reaching as high as 98% in July, September and in December 2020, as compared to 32% in January and 41% in February 2020.

**Table 1:** measles reported cases and specimens collected through the measles case-based surveillance system 2014-2020 (WHO African Region)

Parameter	Measles reported cases and specimens collected through the case based surveillance system, 2014–2020. WHO African region						
	2014	2015	2016	2017	2018	2019	2020
**Total suspected measles cases reported through the case-based surveillance system**	71566	68769	61771	56582	82805	323424	70242
**Laboratory specimens collected from suspected measles cases**	42320	38249	39450	46027	58328	61632	39613
**Laboratory confirmed rubella cases**	6106	5546	3450	4633	3523	5156	2848
**Laboratory confirmed measles cases**	8329	6717	6009	4963	7534	15820	11781
**Total measles cases confirmed by laboratory, epidemiological linkage and clinical compatibility**	37847	37938	28823	16940	45842	281270	44512
**% measles IgM positive among those tested**	16%	21%	18%	11%	16%	27%	31%
**Regional incidence of confirmed measles per million population**	40	39.4	29.1	16.9	43.3	255	39.7

**Table 2:** change in the number of suspected measles cases reported and specimens collected in 2020 as compared the national average for 2014-2018 (WHO African Region)

Country	Mean number (and range) of suspected measles cases for 2014-2018	Number of suspected measles cases in 2020	Percentage change in number of suspected cases in 2020 as compared to the mean for previous years	Mean number (and range) of specimens collected for 2014-2018	Number of specimens collected in 2020	Percentage change in number of specimens collected in 2020 compared to the mean for previous years
Algeria	182 [49-345]	101	-45%	166 [48-345]	96	-42%
Angola	2772 [258-12315]	1414	-49%	536 [258-1134]	719	134%
Benin	592 [234-1072]	226	-62%	492 [230- 910]	226	-54%
Botswana	291 [112-923]	41	-86%	283 [109-899]	40	-86%
Burkina Faso	710 [158-2008]	1857	261%	710 [158-2005]	1857	262%
Burundi	188 [142-303]	1968	1045%	188 [142-303]	504	268%
Cameroon	1973 [1560 - 2770]	1963	-1%	1604 [1323-1868]	1189	-26%
Cape Verde	1 [0-3]	1	0%	0 [0-2]	0	0%
Central African Republic	476 [231-743]	3692	776%	475 [230-743]	765	161%
Chad	829 [325-1457]	2324	280%	531 [324-539]	544	103%
Comoros	20 [0-48]	41	209%	20 [0-48]	40	200%
Congo	690 [200-1594]	141	-80%	439 [200-978]	141	-68%
Cote d'Ivoire	1367 [623-2239]	2650	194%	1367 [623 - 2239]	2606	191%
D. R. Congo	6221 [3769-7158]	17185	276%	2967 [ 2174 - 3226]	4391	148%
Equatorial Guinea	589 [58-1726]	107	-82%	74 [13-151]	106	144%
Eritrea	194 [103-334]	132	-32%	194 [102-334]	130	-33%
Eswatini	76 [50-100]	47	-38%	75 [50-100]	47	-38%
Ethiopia	1006 [3839-19734]	3423	-66%	3725[2871-5424]	2450	-34%
Gabon	715 [109 - 1719]	81	-89%	714 [109-1719]	81	-89%
Gambia	76 [60-94]	65	-14%	73 [57-94]	64	-13%
Ghana	1468 [1030-2097]	1796	122%	1468 [1030-2097]	1796	122%
Guinea	642 [48-1268]	816	127%	640 [48-1268]	816	128%
Guinea Bissau	0 [0-2]	0	0%	0 [0-2]	0	0%
Kenya	1188 [544-1621]	910	-23%	986 [518-1401]	406	-59%
Lesotho	217 [77-523]	107	-51%	213 [64-523]	106	-50%
Liberia	777 [3-2545]	875	113%	352 [3-1149]	433	123%
Madagascar	5542 [ 366-24808]	579	-90%	955 [ 366-1873]	567	-41%
Malawi	438 [ 233-1057]	60	-86%	433 [233 - 1044]	59	-86%
Mali	671 [266 - 1398]	683	102%	630 [266 - 1398]	683	108%
Mauritania	64 [37 - 120]	36	-44%	64 [37 - 120]	0	-100%
Mauritius	305 [0 - 1525]	0	-100%	305 [0 - 1525]	0	-100%
Mozambique	1180 [446-1800]	2447	207%	1177 [444-1798]	2445	208%
Namibia	812 [2-2077]	219	-73%	727 [2-2029]	219	-70%
Niger	990 [513-1683]	1238	125%	990 [513-1681]	1237	125%
Nigeria	1472 [ 9343-19965]	15176	103%	9071 [5903-11604]	8434	-7%
Rwanda	688 [427-1164]	842	122%	685 [427-1146]	842	123%
Sao tome and Principe	0 [0-1]	1	100%	0 [0-1]	0	0%
Senegal	730 [358 - 998]	573	-22%	729 [358-985]	573	-21%
Seychelles	196 [0-968]	112	-43%	196 [0-967]	112	-43%
Sierra Leone	297 [121-536]	37	-88%	295 [121-525]	37	-87%
South Africa	3341 [1902 - 5288]	1153	-65%	3301 [1706-5287]	1140	-65%
South Sudan	649 [267-1119]	1326	204%	273 [193-371]	194	-29%
Tanzania	1469 [1016-2063]	2481	169%	1363 [929-1960]	2428	178%
Togo	415 [352-682]	205	-51%	415 [295-682]	205	-51%
Uganda	2286 [1498 - 3658]	606	-73%	2080 [1232-3422]	426	-80%
Zambia	381 [ 298-485]	237	-38%	380 [298-484]	237	-38%
Zimbabwe	805 [327 - 2081]	269	-67%	801 [327-2076]	269	-66%

**Trends in measles surveillance performance:** the annual target of 80% districts reporting suspected measles cases was attained at Regional level in all the years except in 2014. In 2020, at the Regional level, the figure was 86%, with 30 (64%) out of the 47 countries in the Region attaining the 80% annual district reporting target. The non-measles febrile rash illness rate (NMFRI rate) has been above the minimum of 2 per 100,000 population at Regional level from 2018 to 2020. However, the lowest rate was recorded in 2020. The number of countries that have attained the target for the NMFRI rate was also the lowest in 2020, with 18 (38%) out of the 47 countries. In comparison, 27 countries met this target in the years 2014-2019. Only 11 countries (23%) met the targets for both principal surveillance performance indicators in 2020 as compared to an average of 21 countries in the years 2014-2019 (Table 3 and Table 4). The annual non-measles febrile rash illness rate declined by more than 50% in 16 countries in 2020 as compared to the average for the years 2014-2019, while 12 countries attained rates above their previous average (Table 4). Similar declines were also noted in the proportion of specimens arriving at the national laboratory within 3 days of collection, falling from an average of 41% in 2014-2019 to 30% in 2020. The proportion of laboratory results sent out timely to the national immunization program level also fell from an annual average of 68% in 2014–2019 to 55% in 2020 (Table 5).

**Table 3:** annual measles case-based surveillance performance 2014-2020 (WHO African Region)

Parameter	Annual surveillance performance						
	2014	2015	2016	2017	2018	2019	2020
% cases that have benefited from investigation including collection of laboratory specimens ≥ 80%	85%	82%	86%	97%	94%	81%	83%
% districts reporting ≥ 80%	77%	82%	82%	82%	88%	89%	86%
Non-measles Febrile Rash Illness (NMFRI) rate ≥ 2.0 per 100,000 population]	3	2.5	2.5	3.1	2.8	3.2	2.1
Number of countries with at least 80% districts reporting	28	24	28	30	33	35	30
Number of countries with NMFRI rate of 2 or more per 100,000 population	26	27	25	30	29	27	18
Number of countries meeting targets for both performance indicators	20	19	20	26	23	22	11
Total number of countries implementing case-based surveillance for measles	44	44	44	44	46	47	47

**Table 4:** surveillance performance in 2020 as compared to the national average for 2014-2019 (WHO African Region)

Country	Surveillance performance in 2020 as compared to the national average for 2014-2019					
	Non-measles febrile rash illness rate (NMFRI)- ≥ 2.0			% districts reporting suspected cases with blood specimen-≥ 80%		
	Average for 2014 - 2019	Range for 2014 - 2019	2020	Average for 2014 - 2019	Range for 2014 - 2019	2020
Algeria	0.1	[0 - 0.2]	0.2	62%	[42 - 100%]	96%
Angola	1.3	[0.8 - 1.9]	1.1	71%	[41 - 100%]	92%
Benin	1.6	[0.9 - 2.1]	1.6	85%	[75 - 100%]	85%
Botswana	12.3	[4.8 - 39]	1.6	85%	[80 - 96%]	76%
Burkina Faso	1.1	[0.6 - 2.0]	1.5	87%	[77 - 100%]	88%
Burundi	1.7	[1.3 - 2.8]	2.5	74%	[64 - 84%]	71%
Cameroon	4.2	[2.5 - 5.8]	1.8	89%	[86 - 91%]	82%
Cape Verde	0.0	[0.0 - 0.0]	0.0	18%	[0 - 100%]	0%
Central African Republic	6.7	[3.7 - 11.9]	4.7	79%	[69 - 100%]	77%
Chad	1.6	[0.4 - 2.4]	0.9	96%	[86 - 100%]	82%
Comoros	6.1	[0.1 - 18.8]	4.5	82%	[0 - 100%]	85%
Congo	4.8	[3.1-7.1]	1.8	92%	[83 - 100%]	96%
Cote d'Ivoire	6.1	[2.3 - 8.8]	7.6	82%	[79 - 87%]	74%
D. R. Congo	2.0	[1.5-2.4]	2.3	63%	[39 - 85%]	82%
Eq. Guinea	2.4	[0.2-6.8]	2.4	68%	[28 - 85%]	60%
Eritrea	3.8	[2.1 - 6.8]	3.1	46%	[29 - 60%]	29%
Eswatini	7.5	[2.7 - 10.3]	3.9	87%	[80 -100%]	80%
Ethiopia	2.2	[1.9 - 2.5]	1.4	91%	[80 - 99%]	84%
Gabon	6.6	[0.5- 14.4]	3.2	79%	[61 - 100%]	86%
Gambia	3.0	[0.9 - 5.3]	2.0	74%	[56 - 100%]	78%
Ghana	3.2	[2.6 - 3.7]	5.5	100%	[100 - 100%]	90%
Guinea	3.8	[0.2 - 6.1]	2.3	83%	[39 - 100%]	84%
Guinea Bissau	0.0	[0.0 - 0.1]	0.0	2%	[0 - 9%]	0%
Kenya	1.8	[1.1 - 2.8]	0.6	87%	[66 - 100%]	90%
Lesotho	11.0	[3.2 - 27.0]	5.2	92%	[ 90 - 100%]	100%
Liberia	4.2	[0.1 - 13.7]	0.0	54%	[6 - 94%]	94%
Madagascar	3.6	[1.5 - 4.7]	1.8	89%	[65 - 100%]	93%
Malawi	2.4	[0.6 - 6.6]	0.3	74%	[52 - 96%]	52%
Mali	1.8	[1.1 - 2.8]	1.0	94%	[79 -100%]	92%
Mauritania	1.4	[0.9 - 3.0]	0.0	59%	[28 - 100%]	0%
Mauritius	0.0	[0.0 - 0.0]	0.0	20%	[ 0 - 0%]	0%
Mozambique	4.8	[3.9 - 5.1]	5.2	99%	[97 - 100%]	98%
Namibia	12.6	[7.3 - 45.6]	7.8	83%	[73 - 100%]	76%
Niger	1.0	[0.6- 2.4]	1.8	99%	[98 - 100%]	97%
Nigeria	2.7	[1.4 - 4.4]	2.3	93%	[81 - 100%]	99%
Rwanda	7.3	[3.6 - 10.4]	6.0	95%	[94 - 97%]	97%
SaoTome and Principe	0.2	[0.0 - 0.0]	0.0	50%	[0 - 0%]	0%
Senegal	4.2	[2.2 - 6.8]	1.8	94%	[89 - 100%]	96%
Seychelles	0.4	[0.0 - 1.2]	8.6	56%	[ 0 - 100%]	5%
Sierra Leone	2.6	[0.9 - 6.2]	0.3	92%	[87 - 93%]	93%
South Africa	6.6	[2.3 - 9.2]	1.9	87%	[78 - 100%]	82%
South Sudan	1.7	[0.8 - 3.1]	0.9	39%	[29 - 49%]	41%
Tanzania	2.0	[0.8 - 2.5]	2.4	97%	[87 - 100%]	90%
Togo	3.8	[1.2 - 7.5]	1.4	96%	[92 - 100%]	96%
Uganda	2.9	[1.9 - 6.8]	0.7	94%	[92 - 100%]	93%
Zambia	1.7	[0.4 - 2.7]	0.9	73%	[53 - 93%]	54%
Zimbabwe	6.0	[2.8 - 15.5]	1.9	93%	[77 - 90%]	92%

**Table 5:** national measles laboratory performance 2014-2020 (WHO African Region)

Parameter	National measles laboratory performance. African Region. 2014-2020.						
	2014	2015	2016	2017	2018	2019	2020
% specimens received at the national measles laboratory within 3 days of collection ≥ 80%	44.8%	43.9%	41.9%	42.0%	36.9%	36.2%	30.0%
% IgM results sent out within 7 days of receipt of specimens ≥ 80%	67.6%	70.5%	68.7%	69.5%	64.4%	65.4%	55.2%
% of countries which attained 80% specimens arriving at the laboratory within 3 days of collection	14.0%	16.3%	11.9%	14.3%	13.6%	9.0%	8.7%

## Discussion

Countries in the African Region of the WHO continue to have wide gaps in measles vaccination coverage and the incidence of measles has remained high [14]. Considering the overall performance trends, the Region did not meet the measles elimination targets for 2020. With the decline in routine immunization services and the postponement of measles Supplemental Immunization Activities (SIAs) in 2020, the risk of countries experiencing big outbreaks was quite significant, indicating the need to maintain high quality surveillance during the period. However, there were no major measles outbreaks reported in the Region in the months after the declaration of the, as compared to the documented outbreaks of 2018 and 2019 [15,16]. This can be due to the gaps in surveillance reported in this study, the closure of schools, as well as the limitation in travel and population movements imposed in response to the COVID-19 pandemic. Another reason is the reduction in the remaining pool of susceptible populations to sustain large outbreaks following the large measles outbreaks of the previous years. As shown in this analysis and in previous studies, measles surveillance performance across the 47 countries in the Region has not shown significant improvement since 2014 in terms of the attainment of the set performance targets [17]. The proportion of the countries that have met the targets for the two main performance indicators has not surpassed more than three fourths at any time between 2014 and 2020. The COVID-19 pandemic posed further threat to the continuity of measles surveillance in 2020. Even though at Regional level the overall annual number of reported cases and collected specimens is comparable to the preceding years, the monthly breakdown indicates a significant and sustained decline in the reported cases and collected specimens after the pandemic hit. In 26 countries, the reported number of suspected cases declined, while 27 countries had lower number of specimens collected in 2020 as compared to the mean for the previous years. The increased number of cases and specimens in 15 countries should be seen in light of the measles incidence and the non-measles febrile rash illness rates, which indicate that, in Central African Republic, Chad, Guinea and Liberia, confirmed measles outbreaks were the major drivers of increased reporting and specimen collection. The overall regional increase in lab confirmed measles cases documented in 2020 is mostly as a result of the outbreaks these countries, and in the Democratic Republic of Congo in early 2020 which continued from the previous year.

In addition, the quality of measles surveillance across the Region in the year 2020 has shown significant decline in terms of the overall sensitivity, with the lowest NMFRI rate and the lowest number of countries attaining the target as compared to previous years. The relatively high proportion of specimens collected, as well as the decline in cases and specimens in the months after the pandemic suggests that the major focus in many countries was investigating measles outbreaks rather than detecting and investigating febrile rash illnesses. The findings in this study are somehow similar to the situation during the 2014-2015 Ebola outbreak of West Africa, when Liberia, Guinea and Sierra Leone all experienced a significant reduction in the number of reported acute flaccid paralysis (AFP) cases over these two years. Liberia failed to meet the minimum target for the non-polio Acute Flaccid Paralysis rate in 2014 and 2015, while Sierra Leone´s performance declined below the target in 2015 [18,19]. Similarly, in the first few months of the COVID-19 pandemic, countries in the European Region of the WHO experienced a decline in measles case reporting [10] Kunjock et al documented a decline in the measles laboratory performance in South Sudan in 2020, especially affecting the time interval between the receipt of specimens and the turnaround of laboratory results [20]. Currently, the collection of blood specimens is the standard for measles IgM serological testing across the Region. In many countries, in order to optimally use available resources, the transportation of specimens to the national serological laboratory in reverse cold chain is done after batching specimens collected within a district over a period of a week or more. In resource constrained areas in general, and in instances when the collection and transportation of specimens becomes problematic, as is the case during this pandemic, the establishment of decentralized serological laboratories, the deployment of filter paper or other easier specimen collection and transport mechanisms, or even point-of-care tests would help to address such challenges and potentially help timely detection and response to outbreaks of measles [21,22]. As countries raise their immunization coverage rates and progress from a high incidence setting to a low incidence setting, the implementation of more stringent surveillance and laboratory standards will be critical to identify smaller outbreaks and stop chains of transmission [23].

**Limitations of the study:** we used reported case-based surveillance data and measles laboratory data for this analysis. During large outbreaks, countries revert to aggregate reporting of suspected measles cases, temporarily suspend collecting laboratory specimens from areas in outbreak status, and confirm cases by epidemiologic link in order not to overload the system. This results in differences between the reported cases in the case-based surveillance system and the aggregate reports. We utilised the two principal surveillance performance indicators for this analysis. However, the monitoring of surveillance performance at country level is done using a variety of indicators measuring the various processes and steps involved in case notification, investigation and reporting. Under-reporting of measles cases from remote areas, and mild measles cases is a known problem in the surveillance system. The incompleteness of reported data, and incomplete processing of laboratory specimens as a result of stock out of laboratory test reagents lead to cases that remain unclassified. In addition, the results from countries with large populations affect the overall Regional performance.

## Conclusion

The overall quality of measles surveillance has declined during the COVID-19 pandemic in many countries in the African Region. However, this decline was not seen in all countries. Countries with surveillance gaps should implement immediate and proactive measures to revitalise active surveillance for measles. While responding to COVID-19 and/ or other communicable disease emergences, countries should continue to closely monitor surveillance performance at national and subnational levels, alongside other efforts to maintain essential services, in order to identify surveillance gaps timely, and implement solutions to address them. In addition, the adoption of decentralised specimen collection and testing methods, as well as considering the establishment of testing laboratories closer to the point of service delivery is recommended to improve timely investigation and prompt confirmation of suspected measles cases, especially in contexts and geographic areas where the transportation of specimens may be challenging.

### What is known about this topic

- The COVID-19 pandemic and the restrictive control measures implemented in many countries have led to some disruption of health services in many countries;
- World Health Organisation provided guidelines for the continuity of essential health services, including surveillance of vaccine preventable diseases.

### What this study adds

- Countries in the African Region of the WHO experienced a significant reduction in measles case reporting and specimen collection in the months following the pandemic, starting April 2020;
- In 2020, fewer countries met the targets for the surveillance performance indicators than in previous years, especially the indicator for the sensitivity of surveillance;
- Countries should take immediate measures to improve measles surveillance performance.
